# Effectiveness of Sound-Based Interventions for Improving Functional Outcomes in Children: A Systematic Review of the Evidence

**DOI:** 10.1155/oti/1693722

**Published:** 2025-06-05

**Authors:** Vanessa Vincent, Gemma Skaczkowski, Donna Hughes-Barton, Kate M. Gunn

**Affiliations:** IIMPACT in Health, Department of Rural Health, Allied Health and Human Performance, University of South Australia, Adelaide, South Australia, Australia

**Keywords:** auditory, listening therapy, sensory processing, sound-based intervention, therapeutic listening

## Abstract

**Introduction:** The aim of this review was to examine the evidence for sound-based interventions in decreasing auditory hypersensitivity and improving behavioural and emotional regulation among children.

**Methods:** A systematic review was conducted of primary research examining the use of sound-based interventions to reduce auditory hypersensitivity and regulate emotion or behaviour. Studies were eligible if they were published in English in a peer-reviewed journal, from January 2010 to March 2023. Studies of any design were included.

**Results:** A total of 4741 titles were identified in the search. Eight papers were eligible, covering six different sound-based interventions. Preliminary evidence highlights improvements in depression and anxiety symptomatology and improved behavioural outcomes including language, listening, and social behaviours. Heterogeneity was high across studies; they varied widely in sample size, population type, study design, and outcomes measured.

**Conclusions:** There is preliminary evidence for the effectiveness of sound-based interventions in improving auditory sensitivity and emotional and behavioural outcomes. However, there is a need for higher quality studies, including randomised controlled trials, and consistency in outcomes across studies to enable a clearer assessment of the evidence.

## 1. Introduction

Occupational therapists commonly work with people who experience behavioural and emotional regulation difficulties, which impact their ability to participate in activities of daily living. Treatment strategies generally use a “top-down” approach whereby the client is engaged in a task-specific activity to achieve their functional goal [1]. Conversely, “bottom-up” approaches focus on performance components, such as balance or strength, to improve participation in the goal [2]. When developing a therapeutic plan for a client, a combination of approaches may be beneficial [2]; as once underlying physiological challenges are addressed and regulation is improved (using “bottom-up” approaches), the client may be more receptive to “top-down” therapies to achieve a specific functional goal [1].

One category of “bottom-up” approaches is sound-based interventions, in which clients use headphones to listen to music which has been modulated [3]. The modulation of music aims to reduce auditory processing challenges and improve concentration [4]. One sound-based intervention currently used by occupational therapists in Australia is the Safe and Sound Protocol (SSP) [5]. Designed as an exercise model for the middle ear muscles, SSP uses the changing frequency band of human prosody to reduce auditory sensitivities and constant sympathetic hyperarousal [6, 7].

SSP is informed by polyvagal theory, which is a hierarchical model that describes the functions of an autonomic nervous system (ANS) and emphasises the importance of felt safety (neuroception) [8]. The theory describes the evolutionary development of three dynamic circuits of the ANS that support different adaptive behavioural strategies in order to regulate the internal state: social communication (e.g., facial expressions, listening, and vocalisations), mobilisation, and immobilisation [9]. According to the theory, “neuroception” describes the neural process that enables people to distinguish safe from dangerous contexts and therefore permit engagement in social behaviours in safe contexts and nonengagement in dangerous ones. In the social communication circuit, during the neuroception process, the viscera (including the heart) are regulated via the myelinated vagus nerve. Therefore, when a context is perceived as safe, vagal influence is increased, heart rate is calmed due to sympathetic influences to the heart being inhibited, and the individual can engage in safe social interactions, including therapeutic interventions. In some clinical conditions, this neural process is disrupted, the heart is influenced by vagal withdrawal, and “safe” environments are perceived as dangerous. As a result, the person is agitated, and the social engagement system is interrupted. In this context, the person's physiological state will not be in an optimal condition for full social, psychological, or behavioural experiences, including learning [8, 9].

According to polyvagal theory, there is an evolutionary connection between heart function and the muscles of the face (including the muscles of listening, facial expression, and vocalisation). It follows that, in a therapeutic situation, addressing underlying performance component deficits such as middle ear dysfunction and auditory hypersensitivities with “bottom-up” sound-based therapies, for instance, may add another facet to aid treatment success when using “top-down” approaches. Measuring physiological outcomes associated with heart function could provide an indicator of the underlying physiological state and a basis on which to detect change in emotional, functional, and behavioural outcomes following an intervention [9].

An earlier version of this type of therapy was called the Listening Project Protocol (LPP) or the Integrated Listening System (iLS) [10]. There are also other sound-based interventions that have been used in clinical practice and explored in the literature, including auditory integration training (AIT) [11], the Tomatis Method [12], The Listening Program (TLP) [13], and therapeutic listening [14].

There is a need for stronger evidence on the impact of sound-based interventions to support their inclusion in current practice, with previous reviews reporting mixed results. Reviews of AIT and the Tomatis Method found that overall, there was no evidence of improvement over control conditions for behavioural problems, cognitive ability, sound sensitivity, or language [4, 15]. Conversely, a systematic review by Villasenor and Smith [16] on the use of sound-based interventions for children with sensory processing challenges found limited evidence for the use of such interventions in improving visual motor, fine motor, communication, and social skills. Other reviews of the Tomatis Method [17] or Therapeutic Listening [18] have also found positive effects of the therapies on a variety of measures of linguistic, psychomotor, cognitive, auditory, social, and emotional skills. More recent reviews focussing on AIT, iLS, TLP, Therapeutic Listening, and frequency modulation devices have identified limited positive benefits to educational outcomes for children with sensory or auditory processing difficulties [16, 19]. However, most of these reviews have focused on specific interventions, outcomes, or populations, and none of these reviews included studies assessing SSP.

## 2. Aims

As noted by other researchers, sound-based interventions are being increasingly used in the clinical setting; however, there is a need for more empirical literature to support their use [20]. The aim of this review was to summarise the evidence for sound-based interventions in decreasing auditory hypersensitivity and improving behavioural and emotional regulation in children. Given the suggestion that behavioural regulation and auditory processing difficulties are linked and that the social engagement system (in particular, the function of the middle ear muscles) can have an impact on the ANS, this systematic review aimed to investigate the evidence of sound-based interventions in improving functional outcomes.

## 3. Method

### 3.1. Study Design and Registration

These Preferred Reporting Items for Systematic Reviews and Meta-Analyses (PRISMA) guidelines [21] guided the reporting of this review. This review protocol was prospectively registered in the Open Science Framework (10.17605/OSF.IO/H57MV).

### 3.2. Eligibility Criteria

This review included primary research focusing on the use of sound-based interventions, which use digitally produced/altered music to reduce auditory hypersensitivity and regulate emotion or behaviour in children or adults. Studies of any design were eligible. Studies were eligible if they were published in English, in a peer-reviewed journal, from January 2010 to March 2023. We excluded theses, letters, opinion pieces, editorials, grey literature, conference abstracts, and review articles, as well as studies addressing music therapy rather than sound-based interventions. We sought to review studies assessing auditory hypersensitivity and emotional or behavioural regulation. Studies addressing the more physiological aspects of hearing, such as loudness perception or hearing ability, were excluded. Additionally, touch or somatosensory interventions, such as vibroacoustic therapy, were excluded, as the focus was not a sound-based intervention.

### 3.3. Search Strategy

The search was conducted in March 2023. Covidence was used for review management [22]. The following databases were searched: Medline (Ovid), Scopus, PsycINFO (Ovid), and ERIC (ProQuest). Additionally, reference lists of the eligible papers were searched for additional studies.

In general, the searches were based around the following key concepts: SSP, iLS, ANS regulation, sensory processing, emotional regulation, and Stephen Porges (author). Searches were adapted for the requirements of each database (see Supporting Information 1 for search strategies). MESH terms or subject headings were used in the Medline, PsycINFO, and ERIC databases, to capture any material that may not have fit the keyword search. The Scopus search was confined to the core keyword search due to an excessive number of titles resulting from a broader search.

### 3.4. Study Selection

Titles and abstracts were screened primarily by one reviewer (VV). Seven hundred and eighty-eight titles/abstracts (17%) were double screened by another reviewer (GS) to ensure consistency of inclusion and exclusion criteria. Full-text screening was conducted by two reviewers (VV and GS). Disagreements at both stages were minimal and easily resolved by discussion (proportionate agreement 92% title/abstract review and 94% full-text review).

Search terms were intentionally broad and did not limit to either children or adults given the anticipated lack of literature in this area. However, the full-text review identified two main categories of sound-based interventions: HIRREM and the SSP (and the earlier iLS and LPP). HIRREM is a noninvasive approach that uses closed-loop acoustic stimulation neurotechnology to facilitate autocalibration of oscillatory patterns [23]. HIRREM requires attaching sensors to the client's scalp for the duration of the session, and, with the exception of Tegeler et al. [24] which included teenagers, all HIRREM studies included in this review were conducted with adults. In contrast, all but one of the remaining studies [6] were conducted with children, with one involving children and young adults to age 21 years [25]. Due to fundamental differences in the HIRREM procedure compared to other sound-based interventions, the current summary was limited to a review of sound-based interventions for children. Studies examining sound-based interventions among adults are summarised in Supporting Information 2.

### 3.5. Data Extraction

Data for each paper were extracted by a minimum of two reviewers (VV, DHB, and GS). A template for data extraction was pilot tested by two reviewers (VV and GS) on five papers and modified as necessary. Data were extracted on study aims, study design, intervention characteristics, outcome measures, participant details, and intervention effectiveness. Findings are synthetised according to the type of intervention. Due to the heterogeneity in study design and outcomes, a narrative synthesis was performed.

## 4. Results

**Figure 1:**
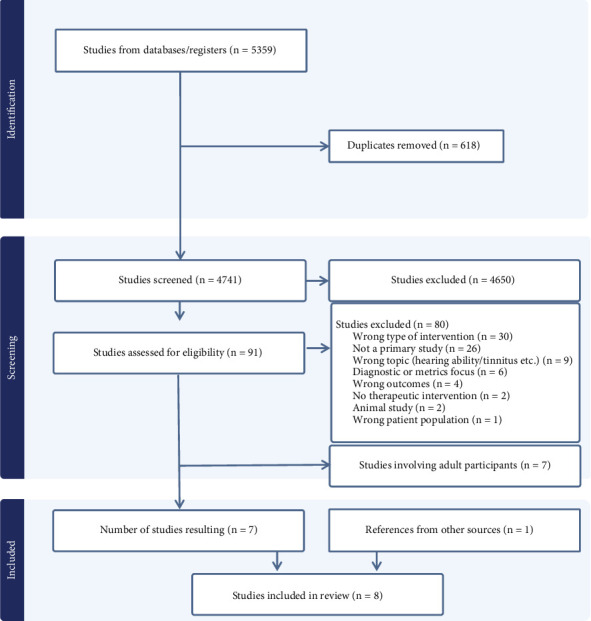
PRISMA flowchart.

The search identified 5359 papers, with removal of duplicates leaving 4741 titles and abstracts for review (see Figure 1). Ninety-one full texts were reviewed, and 11 of these were identified as eligible. Four additional papers were identified through backward and forward citation searching of the eligible studies. Seven of these studies involved adult populations and are summarised in Supporting Information 2 [6, 23, 24, 26–29]. A total of eight papers examined these interventions among children and were included in the current paper.

Six sound-based interventions were identified in the search: two papers focused on SSP [30, 31], two on LPP [7, 25], and one each on iLS [32], filtered music [33], TLP [34], and Therapeutic Listening [35].

Studies covered a range of different participant populations. Eight studies were conducted with children/adolescents who had received the following sensory or behavioural diagnoses: autism spectrum disorders (ASDs) [7, 25, 30, 34], functional neurological disorders [31], sensory processing impairments [32], developmental disabilities [35], and mild-to-moderate developmental delays in children exposed prenatally to cocaine [33].  Table 1 lists each of the identified interventions and a summary of the data extracted from each paper.

### 4.1. SSP

SSP involves listening to vocal music which is digitally filtered to remove low and high frequencies and modulate the “frequency bandwidth associated with the human voice from 50 Hz to 3000 Hz” (p. 3) [6]. SSP is an easily portable and commercially available product which is delivered via an app used over ear headphones [5].

The review retrieved two single case studies that examined the effect of SSP with children: a 20-month-old diagnosed with ASD [30] and a 10-year-old child diagnosed with functional neurological disorder [31]. Results from both studies indicated that SSP was associated with a significant increase in emotional self-regulation. The studies also reported improvements in observed language, listening and processing, and facial expressions [30] and reductions in self-reported depression, stress, anxiety, suicidal thoughts, greater physical abilities, and less frequent seizures [31].

### 4.2. LPP

Two studies examined the LPP [7, 25]. The LPP involves listening to vocal music which had been digitally modified to exaggerate the features of human speech. Both studies focused on the efficacy of LPP for children with ASD, although Porges et al. [25] also included adolescents and young adults up to the age of 21 years. In both studies, the intervention involved daily sessions of LPP for 5 days. Outcomes were assessed pre- and postintervention. One was a randomised controlled trial in which children were randomised to receive filtered music versus no music or filtered music versus unfiltered music [7]. Results showed that children receiving filtered music performed significantly better than children wearing headphones (no music) on measures of hearing sensitivity, spontaneous speech, listening, and behavioural organisation and children receiving filtered music versus unfiltered music performed better on measures of hearing sensitivity and emotional control only. Results from the other study showed a significant improvement pre- and post-LPP on an auditory processing task where children needed to complete a task while filtering through competing word stimuli [25]. However, when postintervention scores from the LPP group were compared to an age-matched control (who had not received LPP), there were no significant differences.

### 4.3. iLS

Another sound-based intervention, iLS, involves listening to acoustically processed, low-frequency music while participating in activities focusing on visual motor, balance, and movement [32]. Schoen and Miller [32] examined the effect of iLS in a small group of seven children aged 5–12 years diagnosed with sensory processing impairments. The children received the intervention for up to 16 weeks, participating in 40 one-hour sessions, one session a day, 5 days a week. Outcome measures were individualised for each child and included behaviour ratings and sensory challenges. Results indicated improvements for some of the children in areas of sensory challenge (i.e., reduced auditory arousal), reduced aggression, internalising, anxiety, and depression and an increased ability to perform activities of daily living.

### 4.4. Filtered Music

Filtered music describes music that has undergone a process to amplify it to the range of human speech as well as magnify the changes of these frequencies [33]. Porges et al. [33] conducted a randomised controlled trial with children aged 17–30 months with mild–moderate developmental delays. Children were randomised to receive therapy involving filtered music, unfiltered music, or no music (control). Results showed that listening to any music, filtered or unfiltered, significantly improved expressive language, with a trend towards higher receptive language, when compared to a control group that did not listen to any music. However, there was no change in cognitive ability, and outcomes did not differ between filtered and unfiltered music conditions.

### 4.5. TLP

TLP uses “psycho-acoustically modified classical music targeting certain frequency ranges that claim to impact functional capabilities” (p. 14) [34]. One paper reported a case study of a 7-year-old child with autism who showed improvements after receiving therapy with TLP for 10 weeks [34]. Specifically, the child showed reduced sensory overresponsivity and decreased self-stimulatory behaviour.

### 4.6. Therapeutic Listening

Therapeutic listening uses electronically modified music in conjunction with traditional sensory integration strategies [14, 35, 36]. One paper reported the results of a small study of 15 preschool children with developmental disabilities, some with multiple additional diagnoses, who received Therapeutic Listening once or twice a day (20–30 min per session) for 6–20 weeks in addition to their regular therapy regimes [35]. Children were assessed pre- and postintervention on a range of validated developmental scales, and results showed significant improvements on measures of language, nonverbal intelligence, social behaviour, visuomotor perception skills, and gross and fine motor skills, while no changes were noted on measures of sensory processing and problem behaviours.

## 5. Discussion

This systematic review examined the empirical evidence of sound-based interventions on auditory hypersensitivity and behavioural or emotional regulation in children. Overall, eight eligible papers were identified, with most studies examining the SSP and the earlier iLS and LPP. The results provide preliminary evidence for the positive impact of these interventions, but the review also identified several limitations in the current evidence.

The studies in this review identified improvements in social behaviour, language, and communication following sound-based interventions. However, inconsistencies in results were also identified. For example, the findings from Porges et al. [25] showed no significant difference in postintervention scores when the treatment group was compared to age-matched controls. Further, a diverse range of outcomes were investigated across studies, the studies generally had small sample sizes, and several were single case reports, limiting the strength of this evidence.

Nonetheless, SSP is portable and only requires children to be able to tolerate the headphones, while an adult provides social support and activities to keep the child occupied during the session. Thus, it could be delivered at home with remote support from a therapist, therapist assistant, or at an outreach clinic, as part of a home or school visit. More research is needed to investigate the effectiveness of these interventions. A further question is whether SSP, when delivered at home, could improve the effectiveness of subsequent face-to-face sessions. If so, this may result in more efficient and cost-effective service delivery, as fewer clinic sessions may be required, which may have particular benefits for those in rural settings who face significant occupational therapy workforce shortages [37].

Although there does appear to be promising evidence of the effectiveness of these interventions, this review also identified several gaps in the quality of the evidence available. Only two of the eight studies reported results of randomised controlled trials: one with LPP [7] and one with filtered music [33]. Randomised controlled trials are considered to provide evidence at Level II on the NHMRC hierarchy of evidence [38]. One therapeutic listening study used a pre-post design [35] providing evidence at Level III-2 due to the lack of a comparable control group. Another study examining LPP was primarily a pre-post study, but with control group data available for some outcomes [25]. The remaining four studies examining SSP, TLP, and iLS were single case studies [30–32, 34], classified as Level IV.

For most studies in this review, sample sizes were small, ranging from *N* = 1 to *N* = 146. Small sample sizes may not provide sufficient power to detect changes in behaviour following the intervention. Another limitation of the body of evidence summarised in this review is the variation in populations, outcome measures, and assessment tools, both within and between studies. There was some variability in data collection techniques, with some therapies being conducted in the home and data collected via observation by a parent. In this instance, it is possible that parental reports of improved behaviour may be due, at least in part, to the social support component of the intervention [7].

Further research is also needed to identify the mechanisms of action and address the question of whether improved outcomes are due to the specific features of modified sound or simply expectation bias. There is some suggestion in the findings of this review that features of the protocol itself may have had a positive impact, separate from the modified music. For example, Porges et al. [33] identified that listening to any music, filtered or unfiltered, improved spontaneous speech, behaviour, and hearing sensitivity in children who had been prenatally exposed to cocaine. A number of reasons were suggested for these findings, including that the music selected (Disney movie soundtracks) may have provided sufficient challenge to the middle ear muscles to result in improved functionality even without filtering. Another study by Porges et al. [7] similarly found that children with ASD who listened to filtered music showed improvement across a variety of outcomes when compared to no music, but only performed better on a few outcomes compared to unfiltered music. This suggests that listening to any music may be beneficial, at least to some degree.

### 5.1. Limitations

A key limitation of this review is the inconsistency in terminology used to name and describe these kinds of interventions. To address this issue, we used very broad search terms and conducted backward searches of the study reference lists. We also limited our search to studies published from 2010 onwards, and the Scopus search was restricted to only keywords, due to an excessive number of titles resulting from the broader search. These changes were made to ensure a feasible search size and based on the dates of publication of one of the leading authors in the field, Stephen Porges. However, due to these restrictions, we acknowledge that some studies may have been inadvertently missed in our summary of the literature on this topic.

### 5.2. Implications

Overall, results of the review suggest that sound-based interventions, as “bottom-up therapies,” offer a promising avenue to address behavioural and emotional dysregulation, with most studies examining interventions similar to the SSP. SSP is portable and has potential as an at-home adjunct to traditional therapy. “Bottom-up” approaches such as these may currently be less well used by occupational therapists, perhaps due to evidence that functional goals are more effectively achieved through “top-down” strategies [39]. However, this review provides some support for the hypothesis that “bottom-up” approaches (such as sound-based interventions) may promote greater benefit from “top-down” therapies and should be considered a part of a multidisciplinary package of treatment to support improved occupational performance.

Overall, the results of this review provide preliminary evidence that sound-based interventions can improve social behaviour, language, communication, auditory sensitivity, and emotional outcomes. However, the evidence for SSP and related approaches included numerous case studies and small pre-post trials, with some inconsistencies between studies and a need for higher quality evidence. At this stage, occupational therapists should be mindful of the limitations in the evidence base of the current literature when considering including sound-based therapies into their treatment plans.

## Figures and Tables

**Table 1:** Data extraction for each eligible study.

**Reference** **Country in which study was conducted**	**Participant characteristics** **- Number** **- Mean age** **- % Female** **- Main condition**	**Intervention and setting**	**Session duration and frequency**	**Study design and timing of measures**	**Outcome measures**	**Key findings**
Safe and Sound Protocol (SSP)						

Rajabalee et al. [31] Australia	*N* = 1 Age 10 years Female Functional neurological disorder	Safe and Sound Protocol Lab/clinic	6 weeks 9 × 15–30 min sessions	Case study Measures: 1. Baseline 2. 1 month post	• DASS • Body Perception Questionnaire	Pre- to posttherapy: • Decrease in depression, anxiety, stress, body awareness, supradiaphragmatic (heart/chest/throat) reactivity, and subdiaphragmatic (gut) reactivity (sig not reported) • All scores moved from the clinical range preintervention to the normative range postintervention • The participant improved in her ability to walk without support, climb on play equipment, able to self-regulate anxiety, and practice regulation strategies. Her suicidal thoughts settled and her functional seizures decreased

Squillace et al. [30] United States	*N* = 1 Age 20 months Female Autism spectrum disorder	Safe and Sound Protocol Home based	12 weeks Phase A1: 10 days × 30 min per day Phase A2: 16 days	Single subject ABAB design Baseline A1 = 10 intervention days B1 = 8 weeks withdrawal A2 = 16 intervention days 1. B2 = final withdrawal Measures: 1. Baseline 2. A1, A2 = daily 3. B1, B2 = weekly 4. 3 months post	• Modified from the SSP questionnaire in key areas of interest (parental observation)	Baseline to postintervention: • Significant improvements in language, listening and processing, facial expression, and emotional regulation • Significant decrease in behaviour score Baseline to 3 months postintervention • Significant improvements in language, listening and processing, facial expression, and emotional regulation • Significant decrease in behaviour score

Listening Project Protocol (LPP)						

Porges et al. [25] United States	*N* = 146 Intervention: *n* = 78: (*n* = 33 subset in Study 2 intervention) Age 6–21 years 156.5 ± 46.6 months (for larger *n* = 78 sample) 8 female (10.3%) Age-matched control: *n* = 68 164.2 ± 52.0 months 9 females (13.2%) Autism spectrum disorder	The Listening Project Protocol Lab/clinic	Study 2 intervention subset study: 5 days Daily × 60 min sessions	Pre/post study ⁣^∗^Control group data available for selected outcomes Measures: 1. 1 week prior 2. Postintervention	• RSA • SCAN Test for auditory processing disorder (filtered and competing words)	Baseline to postintervention: • Significant improvement in filtered and competing words • Significant increase in baseline RSA • Preintervention, RSA increased during the SCAN task. Postintervention, RSA did not increase during the SCAN task • Change in RSA from baseline moderated the correlation between IQ and competing word performance preintervention, but not postintervention Postintervention vs. control: • No difference in filtered and competing word tasks

Porges et al. [7] United States	Trial 1 *N* = 64 Trial 2 *N* = 82 Trial 1 Filtered *n* = 36 M age = 55.37 months (SD = 11.42), 11 females (30.6%) No music *n* = 28 M age = 52.67 months (SD = 11.30), 5 females (17.9%) Trial 2 Filtered *n* = 50 M age = 53.33 months (SD = 15.95), 6 females (12%) months Unfiltered *n* = 32 M age = 56.74 months (SD = 9.25), 5 females (15.6%) Autism spectrum disorder	The Listening Project Protocol Lab/clinic	5 days Daily × 45 min	RCT Trial 1: Filtered music vs. headphones only (no music) Trial 2: Filtered vs. unfiltered music Measures: 1. Baseline 2. Postintervention 3. Parent report 1 week post	• Parental report—behaviour questionnaire • Semistructured play-based behavioural assessments • Social Interaction Coding Scale	Filtered music condition vs. headphones only condition: • Filtered music condition showed greater improvement in hearing sensitivity, spontaneous speech, listening, and behavioural organisation • Summed across domains, filtered music group showed significantly more improvements than the headphones only group Filtered music condition vs. unfiltered music condition: • Filtered music group showed greater improvement in hearing sensitivity and emotional control • No differences in the number of improvements summed across domains Filtered music condition: • Sharing behaviours increased, but only for the children with improvement on hearing sensitivity following the intervention

Integrated Listening Systems (iLSs)						

Schoen et al. [32] United States	*N* = 7 5–12 years 3 females (42.9%) Sensory processing impairments	ILS (focus series) Home and clinic	8 weeks (approx. 16 weeks total for study) delivered 5 days 4 times at home and 1 at the clinic 40 × 60 min sessions	Single subject ABA design A = baseline (3–5 weeks) B = 8 week intervention A = return to baseline (2–4 weeks) Measures: 1. Baseline 2. A = weekly 3. B = weekly 4. A = weekly	• Individualised behavioural goals • Sensory Challenge Protocol • Adaptive Behaviour Assessment System-II • Behaviour Assessment System for Children-2	Baseline to postintervention: • Significant decrease in behavioural measures (BASC): Externalising, hyperactivity, aggression, internalising, anxiety, depression, behavioural symptoms, and atypicality • Significant increase in behavioural measures (BASC): Adaptive skills, adaptability, and activities of daily living • Significant increase in communication and self-care (ABAS) • No difference in other subscales of the ABAS • Positive change for 23/28 goals from baseline to intervention that was maintained or increased postintervention • Reduction in electrodermal activity for 4/7 participants • Reduction on two to four sensory challenges for 3/7 participants

Filtered music						

Porges et al. [33] United States	Total *N* = 62 Age: *M* = 20.9 months (SD = 3.7) 32 females (51%) Filtered music *n* = 18 Age, 11 females (61.1%) Unfiltered music *n* = 20 Age, 10 females (50%) Control (initial assessment only) *n* = 24 11 females (45.8%) Moderate developmental delays	Filtered music Clinic	16 weeks 5 days per week First week: 50-min sessions daily Subsequent 15 weeks: 10-min sessions daily	RCT Three conditions: 1. Filtered music 2. Unfiltered music 3. Control Measures: 1. 1 month prior 2. 1 month post	• Reynell Developmental Language Scales • Bayley-II	Filtered vs. unfiltered conditions: • No significant differences in expressive or receptive language, or cognitive ability Filtered + unfiltered vs. control conditions: • No difference between music and control on cognitive ability • Significantly higher expressive language in the music vs. control group • Trend towards higher receptive language in the music vs. control group

The Listening Program (TLP)						

Gee et al. [34] United States	*N* = 1 7 years Female Autism spectrum disorder	The Listening Program Phases A1, A2: At clinic Phase B: At home (with four occasions at clinic—once each in Weeks 2, 5, 8, and 10)	10 weeks 5 days per week, twice per day × 15 min	Single subject ABA design Phases: A1 = baseline response to auditory stimuli (4 weeks) B = 10 week intervention A2 = response to auditory stimuli (4 weeks) Measures: 1. A1 = weekly 2. B = at Weeks 2, 5, 8, and 10 3. A2 = weekly	• Sensory processing measure • Sensory overresponsivity scales Observation of self-stimulatory behaviour	Pre- to postintervention: • Sensory overresponsivity scales: Positive behaviours on the auditory domain trended upward and negative behaviour trended downward • Scores on the hearing subscale and the total sensory processing score tended to show improvement pre- to postintervention and declined during the next postintervention phase • Frequency and duration of self-stimulatory behaviour trended downward pre- to postintervention and increased slightly during the next postintervention phase

Therapeutic listening						

Bazyk et al. [35] United States	*N* = 15 3–6 years 5 females (33.3%) Heterogeneous presentation incl. Limitations in attention, language function, and overall classroom participation	The therapeutic listening program School based	6–20 weeks 5 days per week 1–2 times per day × 20–30 min	Pre/post study Measures: 1. Baseline 2. 6–7 months post	• Peabody Developmental Motor Scales-2 • Developmental Test of Visual-Motor Integration • Draw-A-Person • Preschool Language Scale-3 • Social Skills Rating System • Sensory Profile Questionnaire	Pre- to postintervention: • Significant improvement in language, nonverbal intelligence, social behaviour, visuomotor perception skills, and gross and fine motor skills • No changes in the SSRS problem behaviour score, the PLS-3 language standard score, or the Sensory Profile subtests • Improvements in developmental outcomes (e.g., observational reports of attention, understanding, and participation) (no data provided)

*Note:* Bayley-II = Bayley Scales of Infant Development Mental Scale.

Abbreviations: DASS = Depression Anxiety and Stress Scale; RCT = randomized controlled trial; RSA = respiratory sinus arrhythmia.

## Data Availability

The data that support the findings of this study are available from the corresponding author upon reasonable request.

## Nomenclature

AIT: auditory integration training
ANS: autonomic nervous system
HIRREM: high-resolution, relational, resonance-based, electroencephalic mirroring
iLS: Integrated Listening System
LPP: Listening Project Protocol
SSP: Safe and Sound Protocol
TLP: The Listening Program

## Supporting Information

Supporting Information Additional supporting information can be found online in the Supporting Information section. Supporting Information 1: Search terms for each database. Supporting Information 2: Sound-based interventions in adult populations.
