# Supplementary material for: Effectiveness of Sound-Based Interventions for Improving Functional Outcomes in Children: A Systematic Review of the Evidence
Source: Occup Ther Int. 2025 Jun 5;2025:1693722. doi: 10.1155/oti/1693722 (PMC12163202; doi:10.1155/oti/1693722)
Supplement: Supporting Information — Additional supporting information can be found online in the Supporting Information section. Supporting Information 1: Search terms for each database. Supporting Information 2: Sound-based interventions in adult populations. [file 1693722.f1.docx]

**Supplementary Materials 1. Search Terms for each Database**

**Medline (OVID)**

| **#** | **Query** |
| --- | --- |
| 1 | porges sw.au. |
| 2 | (integrated listening system* or (safe adj3 sound protocol) or listening project protocol or polyvagal theory or stephen porges).ti,ab,kf. |
| 3 | (Reducing Auditory Hypersensitivities in Autistic Spectrum Disorder or "Respiratory Sinus Arrhythmia and Auditory Processing in Autism").rf. |
| 4 | 1 or 2 or 3 |
| 5 | Hyperacusis/ |
| 6 | (sensory processing adj3 (impair* or condition* or problem* or disorder*)).ti,ab,kf. |
| 7 | ((auditory adj3 (sensitiv* or specificity or hypersensitiv*or process* or hyperasthesia* or hyperesthesia* or hyperaesthesia*)) or hyperacusis).ti,ab,kf. |
| 8 | Hyperesthesia/ |
| 9 | ((autonomic or behavio* or state or vagal or self or emotional) adj3 regulat*).ti,ab,kf. |
| 10 | (Social engagement or social behaviour or social outcome* or social participation).ti,ab,kf. |
| 11 | Autonomic Nervous System/ and (retune or re tune or regulat*).ti,ab,kf. |
| 12 | Sympathetic Nervous System/ and (retune or re tune or regulat*).ti,ab,kf. |
| 13 | ((autonomic nervous system or sympathetic nervous system) adj3 (retune or re tune or regulat*)).ti,ab,kf. |
| 14 | Acoustic stimulation/ |
| 15 | ((acoustic or auditory) adj3 stimulat*).ti,ab,kf. |
| 16 | Ear, Middle/ |
| 17 | ((middle ear* or tympanic cavit* or tympanum*) adj3 (therap* or intervention* or trial*)).ti,ab,kf. |
| 18 | Evoked Potentials, Auditory/ |
| 19 | auditory evoked potential*.ti,ab,kf. |
| 20 | Auditory Perception/ |
| 21 | (auditory perception or auditory processing).ti,ab,kf. |
| 22 | ((sound or listen* or auditory or acoustic* or sound*) and (therap* or intervention* or trial*)).ti,ab,kf. |
| 23 | 5 or 6 or 7 or 8 or 9 or 10 |
| 24 | 11 or 12 or 13 |
| 25 | 14 or 15 or 16 or 17 or 18 or 19 or 20 or 21 or 22 |
| 26 | 24 or 25 |
| 27 | 23 and 26 |
| 28 | 4 or 27 |
| 29 | limit 28 to yr="2010 -Current" |

Search yielded *n*= 2437 papers

**PsycINFO (OVID)**

| **#** | **Query** |
| --- | --- |
| 1 | porges stephen w.au. |
| 2 | (integrated listening system* or (safe adj3 sound protocol) or listening project protocol or polyvagal theory or stephen porges).tw. |
| 3 | (Reducing Auditory Hypersensitivities in Autistic Spectrum Disorder or "Respiratory Sinus Arrhythmia and Auditory Processing in Autism").rf. |
| 4 | 1 or 2 or 3 |
| 5 | Hearing Disorders/ |
| 6 | (sensory processing adj3 (impair* or condition* or problem* or disorder*)).tw. |
| 7 | ((auditory adj3 (sensitiv* or specificity or hypersensitiv*or process* or hyperasthesia* or hyperesthesia* or hyperaesthesia*)) or hyperacusis).tw. |
| 8 | ((autonomic or behavio* or state or vagal or self or emotional) adj3 regulat*).tw. |
| 9 | (Social engagement or social behaviour or social outcome* or social participation).tw. |
| 10 | Autonomic Nervous System/ and (retune or re tune or regulat*).tw. |
| 11 | Sympathetic Nervous System/ and (retune or re tune or regulat*).tw. |
| 12 | ((autonomic nervous system or sympathetic nervous system) adj3 (retune or re tune or regulat*)).tw. |
| 13 | Auditory stimulation/ |
| 14 | ((acoustic or auditory) adj3 stimulat*).tw. |
| 15 | Middle Ear/ |
| 16 | ((middle ear* or tympanic cavit* or tympanum*) adj3 (therap* or intervention* or trial*)).tw. |
| 17 | Auditory Evoked Potentials/ |
| 18 | auditory evoked potential*.tw. |
| 19 | Auditory Perception/ |
| 20 | (auditory perception or auditory processing).tw. |
| 21 | ((sound or listen* or auditory or acoustic* or sound*) and (therap* or intervention* or trial*)).tw. |
| 22 | 5 or 6 or 7 or 8 or 9 |
| 23 | 10 or 11 or 12 |
| 24 | 13 or 14 or 15 or 16 or 17 or 18 or 19 or 20 or 21 |
| 25 | 23 or 24 |
| 26 | 22 and 25 |
| 27 | 4 or 26 |
| 28 | limit 27 to yr="2010 -Current" |

Search yielded *n*= 2509 papers

**ERIC (ProQuest)**

((author(porges stephen w) OR ti,ab,if("integrated listening system*" OR (safe NEAR/3 "sound protocol") OR "listening project protocol" OR "polyvagal theory" OR "stephen porges")) OR ((ti,ab,if(("autonomic nervous system" OR "sympathetic nervous system") NEAR/3 (retune OR "re tune" OR regulat*)) OR (ti,ab,if((acoustic OR auditory) NEAR/3 stimulat*) OR ti,ab,if(("middle ear*" OR "tympanic cavit*" OR tympanum*) NEAR/3 (therap* OR intervention* OR trial*)) OR ti,ab,if("auditory evoked potential*") OR (MAINSUBJECT.EXACT("Auditory Perception")) OR ti,ab,if(("auditory perception" OR "auditory processing")) OR ti,ab,if((sound OR listen* OR auditory OR acoustic* OR sound*) AND (therap* OR intervention* OR trial*)))) AND (ti,ab,if("sensory processing" NEAR/3 (impair* OR condition* OR problem* OR disorder*)) OR ti,ab,if((auditory NEAR/3 (sensitiv* OR specificity OR hypersensitiv* OR hyperesthesia* OR hyperaesthesia* OR hyperesthesia* OR hyperaesthesia*)) OR hyperacusis) OR ti,ab,if((autonomic OR behavio* OR state OR vagal OR self OR emotional) NEAR/3 regulat*) OR ti,ab,if("Social engagement" OR "social behaviour" OR "social participation")))) AND pd(20100101-20230302)

Search yielded *n*= 74 papers

**Scopus**

## ( PUBYEAR  >  2009 )  AND  ( AUTHOR-NAME ( porges,  AND  s  AND  w )  OR  ( REFTITLE ( "Reducing Auditory Hypersensitivities in Autistic Spectrum Disorder"  OR  "Respiratory Sinus Arrhythmia and Auditory Processing in Autism" ) )  OR  ( TITLE-ABS-KEY ( "integrated listening system*"  OR  ( safe  W/3  "sound protocol" )  OR  "listening project protocol"  OR  "polyvagal theory"  OR  "stephen porges" ) ) )

Search yielded *n*= 339 papers

**TOTAL = 5,359 papers**

**SUPPLEMENTARY MATERIALS 2. Sound-based interventions in adult populations**

Seven studies examined sound-based interventions in adult populations. One of these studies examined the use of the Safe and Sound protocol; this was a pre-post pilot study conducted with a small group of six adults diagnosed with ASD [6]. Participants received SSP for one hour each day for five consecutive days and completed a battery of self-rated and family-rated assessments immediately before and after therapy. Results identified a significant increase in observed social awareness, but no other significant differences on other subscales of the SRS-2, self- or family-reports (social motivation, social cognition, social communication, restricted interested and repetitive behaviours), or from self-reported sensory profile ratings, symptoms of depression, anxiety or quality of life.

Six studies examined the HIRREM protocol (Brain State Technologies, Scottsdale, Arizona, USA) [23, 24, 26-29]. HIRREM is a non-invasive approach that uses closed-loop acoustic stimulation neurotechnology to facilitate auto-calibration of oscillatory patterns. Brain wave activity is initially assessed and the dominant frequencies identified in this analysis are translated to acoustic stimuli and replayed to the participant via headphones [23]. HIRREM is a clinic-based tool requiring access to specific equipment and technologists who can interpret brain wave assessments. HIRREM also requires attaching sensors to the client’s scalp for the duration of the therapy and assessment session.

Of these six studies, two reported results of randomised controlled trials [26, 29] and four reported results of outcome measurements taken pre- and post-HIRREM therapy [23, 24, 27, 28]. In all studies, HIRREM was conducted over a number of 90-120 minute sessions, ranging from 10 sessions administered over three weeks [29] to 19 sessions administered over 29 days [24].

Results from these studies provide emerging evidence to suggest a positive association between HIRREM and a reduction in clinical symptoms in adults. Five studies identified a significant reduction in insomnia severity following HIRREM [23, 24, 26, 27, 29], and results were maintained up to six months post-intervention [27]. HIRREM was also associated with a significant reduction of self-reported symptoms of post-traumatic stress among a cohort of military personnel, that were maintained six-months post-therapy [27], and self-reported post-concussion symptoms in athletes [24].

Two studies examined the effect of HIRREM on anxiety [27, 29] and all six studies investigated the efficacy of HIRREM on depression. The study measuring anxiety pre-post HIRREM reported a significant reduction in self-reported symptoms of anxiety post-therapy [27]. Five of the six studies assessing depression reported a significant reduction in self-rated depression scores pre-to-post HIRREM [23, 24, 26-28], but results from the randomised controlled trial found no impact of HIRREM on the severity of self-reported anxiety or depression compared to a control group [29].
